# The advances in the regulation of immune microenvironment by *Candida albicans* and macrophage cross-talk

**DOI:** 10.3389/fmicb.2022.1029966

**Published:** 2022-11-18

**Authors:** Shuo Zhao, Anquan Shang, Mengchen Guo, Liangliang Shen, Yu Han, Xin Huang

**Affiliations:** ^1^Department of Dermatology, School of Medicine, Tongji Hospital, Tongji University, Shanghai, China; ^2^Department of Laboratory Medicine, The Second People’s Hospital of Lianyungang, Lianyungang, China

**Keywords:** *Candida albicans*, macrophages, PRR, PAMP, morphological transition, immune escape

## Abstract

Candida albicans (*C. albicans*) is the most common causative agent of invasive fungal infections in hospitals. The body defends against and eliminates *C. albicans* infection by various mechanisms of immune response, and the latter mechanism of immune evasion is a major challenge in the clinical management of *C. albicans* infection. The role of macrophages in combating *C. albicans* infection has only recently been recognized, but the mechanisms remain to be elucidated. This review focuses on the interaction between *C. albicans* and macrophages (macrophages), which causes the body to generate an immune response or *C. albicans* immune escape, and then regulates the body’s immune microenvironment, to explore the effect of *C. albicans* virulence resistance vs. macrophage killing and clarify the role and mechanism of *C. albicans* pathogenesis. In general, a thorough understanding of the molecular principles driving antifungal drug resistance is essential for the development of innovative treatments that can counteract both existing and emerging fungal threats.

## Introduction

**FIGURE 1:**
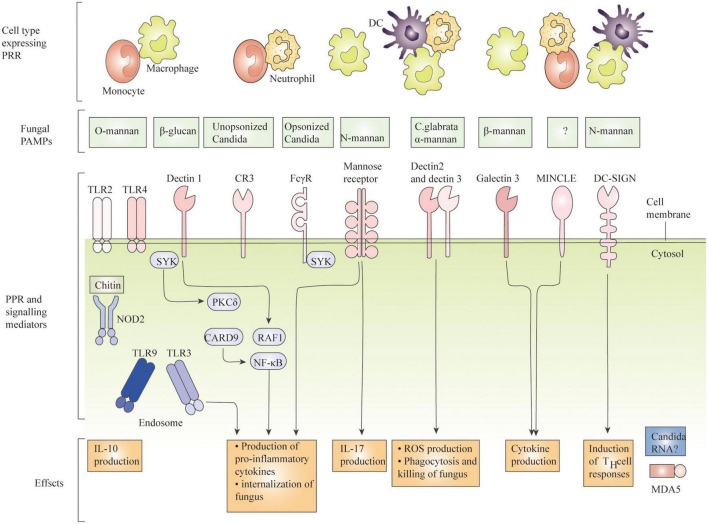
Recognition of *C. albicans* by immune cells.

In recent decades, *Candida albicans* (*C. albicans*) has been the leading cause of life-threatening invasive fungal infections, and despite the low treatment difficulty, invasive candidiasis ranges from mild symptomatic bacteremia to fulminant sepsis with an associated mortality rate of more than 70% (Pappas et al., 2018). According to recent reports, there are approximately 750,000 cases worldwide IC and over 50,000 deaths per year (Gonzalez-Lara and Ostrosky-Zeichner, 2020). *C. albicans* infection is the second most common cause of vaginal candidiasis (VVC). Among women of reproductive age, approximately 75% had at least one episode of VVC, and 40% had a second episode (Rolo et al., 2020). *C. albicans*, an important fungal pathogen in humans, exhibits different morphologies, such as yeast, pseudohyphae, and hyphae, which are recognized differently by the phagocytic cells of the innate immune response. Once *C. albicans* cells get into host tissues, immune cells like macrophages are drawn to the site of infection and activated to find, engulf, and kill the pathogen (Godoy et al., 2022). Understanding the virulence characteristics of *C. albicans*, the tissue-specific mechanisms of anti-Candida host defense, and its resistance mechanisms to existing antifungal agents should lead to better strategies to diagnose and treat affected individuals, which may help improve patient outcomes (Lopes and Lionakis, 2022). Cells of the innate immune system, including N eutrophils (N), macrophages (M), D endritic cells (DC), natural killer (NK) cells, and mast cells, play various roles in innate immune defense (Figure 1). Neutrophils can form neutrophil capture networks to kill pathogens (Jiang et al., 2019), but DC activates antigen-presenting cells and activates initial T cells to kill pathogens (de Albuquerque et al., 2018). Macrophages use surface pattern recognition receptor (PRRs) to recognize and phagocytose *C. albicans* by not only producing reactive oxygen species and reactive nitrogen species but also triggering the activation of signaling pathways such as MAPK and NF-kB, producing pro-inflammatory cytokines, and recruiting other immune cells to work together to eliminate pathogens (Gantner et al., 2005). A study found that *C. albicans* biofilm formation can destroy fine macrophages, and macrophages can also affect biofilm formation (Alonso et al., 2017; Arce Miranda et al., 2019). Candida-macrophage interactions are important immune defense responses associated with disseminated and deep-seated candidiasis in humans (Klotz et al., 2022). During *C. albicans* infection, macrophages can effectively detect, internalize, and kill invading pathogens, while *C. albicans* can escape macrophage killing by different pathways, and the interaction between the two leads to different diseases. And the mechanism by which *C. albicans* escapes lysosome digestion by macrophages remains to be investigated. In this paper, we describe how *C. albicans* identifies the cell wall components of *C. albicans* through the PRRs of macrophages, how macrophages engulf *C. albicans*, how they alter the phagocyte environment to induce hyphal formation, how *C. albicans* and macrophages interact, how *C. albicans* virulence factors, proteins, toxins and GPI proteins are responsible for its virulence on macrophages, etc. To provide a solid foundation for future research on *C. albicans* and macrophages. Understanding the mechanisms by which macrophages interact with *C. albicans* will facilitate more efficient killing of *C. albicans* by macrophages and reduced mortality from invasive *C. albicans* disease.

## *Candida albicans* cell wall, structure and macrophages corresponding pattern recognition receptors

**FIGURE 2:**
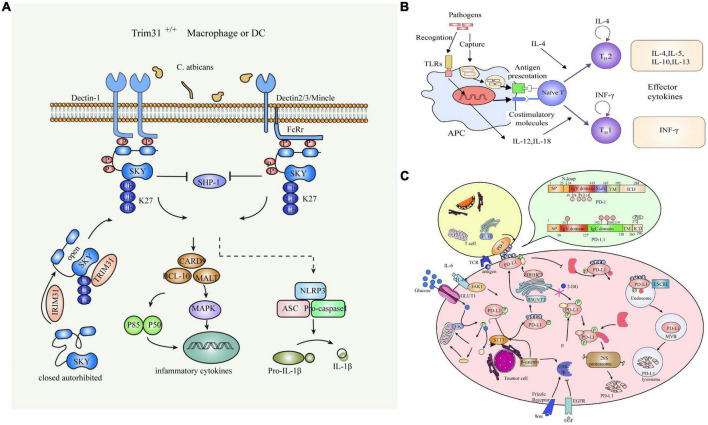
Common PRRs of *C. albicans* interacting with Macrophages. **(A)** Schematic diagram of the C-type lectin receptor. **(B)** Toll-like receptors. **(C)** NOD-like receptors.

The cell wall of *C. albicans* is divided into an inner and an outer layer. The outer layer is mainly composed of mannose and proteins, with mainly O- and N-type mannose polymers (mannose) covalently linked to proteins to form glycoproteins. Among them, O-chain mannan, N-chain mannan and phosphorylated mannan are the main proinflammatory factors, and the inner layer is composed of skeletal polysaccharides, β-1,3-glucan, β-1,6-dextran and chitin, and its main components give shape and survival advantages to the cell (Silao et al., 2020). Compared with other pathogens, fungi can more easily activate the recognition mechanisms of the immune system, and almost all cell wall components belong to PAMP and interact with the corresponding PRRs to elicit an immune response in the host. Common PRRs where *C. albicans* interacts with macrophages mainly include type C lectin receptors (CLRs), Toll-like receptors (TLRs), and NOD -like receptors (NLRBs) (Figure 2).

## Macrophages participate in the regulation of *Candida albicans* the formation of mycelium

There are four forms of *C. albicans*: yeast, hyphae, pseudomycelium, and chlamydial spores. The yeast is more resistant to macrophage killing and its virulence than the hyphal body. The ability of yeast to transform into hyphae is considered one of the most important pathogenic features of *C. albicans*. The transformation of yeast is often, but not always, influenced by environmental conditions such as pH, CO_2_ levels, anaerobic conditions, and temperature. Among others, activation of the CAMP pathway plays an important role in the induction of certain genes. Expression during hyphal formation. Many previous studies have shown that environmental factors such as serum, CO2 concentration, glucosamine N-acetate (GlcNAc), and amino acids can activate the Ras- CAMP -signaling pathway under *in vitro* culture conditions (Salvatori et al., 2020). The CAMP -mediated signaling pathway is a protease. A (PKA) complex activates transcription factors that promote hyphal-specific gene expression and thus hyphal formation.

## Effect of macrophages on *Candida albicans* virulence

*C. albicans* is harmless as a commensal bacterium, but when the balance of normal flora is disturbed or immune defenses are compromised, these fungi can overgrow the mucosal flora and cause symptoms of disease. Two main types of infections have been observed after host immune status is compromised: superficial and invasive candidiasis. Superficial infection of the mucosal epithelium is common in immunocompromised patients, including chronic atrophic stomatitis, chronic cutaneous mucosal candidiasis, and vulvovaginal vaginitis. In more severe cases, *C. albicans* can enter the bloodstream and affect almost all organs of the body. Invasive candidiasis includes either acute or chronic hematogenous disseminated candidiasis and infection of a single or multiple deep organs, either by hematogenous seeding or by direct seeding. Although there is evidence that *C. albicans* can also disseminate in the lymphatic system, the major route of transmission is blood, and once Candida cells invade host tissues, the innate immune response is dominated by macrophages. Phagocytosis of these mononuclear phagocytes has been shown to slow the growth of *C. albicans* and leads to upregulation of genes involved in alternative carbon utilization and stress response. A study found that the importance of stress response pathways in drug resistance and tolerance is clear. These pathways directly affect the ability of the fungus to persist in its environment, and for fungal pathogens in humans, these signaling networks are critical factors in treatment failure (Henriques and Williams, 2020). In phagolysosomes, *C. albicans* is able to transform into a hyphal morphology that allows the pathogen to escape macrophages and thus continue to proliferate in the host. The hyphal-related aspartyl proteases Sap4, Sap5, and Sap6 have been shown to be required for macrophage survival. Proteases and phosphatomannose (PLM) on the surface of *C. albicans* can also promote macrophage survival by inducing apoptosis, for example by interacting with the ERK1/2 signal transduction pathway (Ibata-Ombetta et al., 2003). Some less pathogenic Candida species do not appear to respond in this way and are not viable (Mavor et al., 2005).

## *Candida albicans* interacts with macrophages

As the first line of defense of the innate immune response, macrophages mediate the clearance of invading *C. albicans* by intracellular killing. However, *C. albicans* has evolved sophisticated strategies for targeting macrophages to evade immune surveillance. The cytolytic peptide toxin, Candida lysine, promotes this fungal defense mechanism by disrupting immune cell membranes to escape the phagosomal environment. In the initial phase of interaction between *C. albicans* and macrophages, host cell lysis is mainly involved in mediating caspase-1-dependent cell pyroptosis through the activation of NOD -like receptor protein 3 (NLRP3) (Pietrella et al., 2013; Mao et al., 2020). The cytolytic peptide toxin Candida lysin contributes to macrophage lysis but also activates NLRP3, triggering secretion of pro-inflammatory cytokines that regulate macrophage defense against *C. albicans*. Candida bacteriolysin can be considered a microbial factor that has a dual function during its interaction with the host (König et al., 2020). It provides a mechanism for host cell lysis that contributes to escape from these immune cells. By activating NLRP3, it triggers a host proinflammatory protective response to eliminate *C. albicans*. This combination of beneficial and harmful effects on the host has also been proposed for the role of bacterial pore-forming toxin during its interaction with macrophages. The toxin causes host cell membrane damage associated with *C. albicans* invasion, translocation through the barrier, and escape from phagocytes. Macrophages are essential for the control of disseminated *C. albicans*. Elimination of *C. albicans* is mediated by a combination of antimicrobial activity during phagocytosis. *C. albicans* can counteract these attempts by producing hyphae that induce pyroptosis, mechanically stretching and eventually disrupting the phagosomal membrane, triggering immune cell death (Tucey et al., 2018; Li et al., 2022). However, Candida hemolysin also induces NLRP3 activation, leading to an enhanced host-protective proinflammatory response in mononuclear phagocytes (Kasper et al., 2018). Thus, Candida hemolysin promotes immune evasion by acting as a classical virulence factor but also contributes to the antifungal immune response (Case et al., 2021). Their differential effects in oral, vaginal and systemic infections highlight the dual function of this toxin in the interactions between *C. albicans* and macrophages as classical virulence factors and virulent toxic factors in mucosal and systemic infections (Patterson et al., 2013).

### *Candida albicans* mediates the macrophages immune response

#### Different forms and different parts of *Candida albicans* cause different immune responses

The ability of *C. albicans* to rapidly and reversibly switch between yeast and filamentous morphology is critical to pathogenesis, and the dextrans of the yeast cell wall are mainly protected by epithelial components. The normal mechanism of yeast germination and cell shedding is that adequate exposure *via* dectin-1 results in permanently scarred β-dextrans, including phagocytosis and activated production of reactive oxygen species. Pathogens are also unable to activate dectin-1 in the absence of β-dextran exposure without cell detachment or subsequent exposure during filamentous growth. It is believed that (Hasebe et al., 2018), The form of *C. albicans* directly affects the ability of phagocytes to recognize fungi. The migration of macrophages to *C. albicans* depends on the glycosylation state of the fungal cell wall, which significantly slows the phagocytosis rate of aberrant glycosylated mutants adhering to the macrophage surface, as it is related to the recognition of PAMP by the PRR. In addition, macrophage phagocytosis was significantly faster in hyphae than in yeast cells, and the phagocytosis rate of *C. albicans* hyphae was also affected by spatial arrangement. *C. albicans* contacts macrophages more readily than yeast, and different forms of *C. albicans* elicit different immune responses in macrophages.

#### The effect of macrophages from different sources on *Candida albicans*

Macrophages present in tissues play an important role in controlling disseminated fungal infections. Insufficient accumulation of macrophages in the kidney leads to renal failure and death due to deficiency of the chemokine receptor CX3CR 1. In addition, patients with CX3CR 1 function impaired by a polymorphism are more susceptible to diffuse candidiasis, suggesting an important role of macrophages in the kidney against disseminated Candida infection (Diez-Orejas et al., 2018a). Hematopoietic stem cells and macrophage progenitors differentiate into macrophages to engulf *C. albicans* and produce pro-inflammatory cytokines *via* the TLR2 and MyD 88-dependent pathways.

##### The extracellular sterilization and antibacterial methods of the macrophages

Macrophages not only activate a number of signaling pathways to secrete antimicrobial substances that kill *C. albicans* intracellularly, but also attempt to kill *C. albicans* and inhibit its survival by extracellular means. Macrophages are activated after contact with *C. albicans*. They intercept and kill *C. albicans* by releasing extracellular traps (METs) based on the etosis principle, which trap *C. albicans* at the site of infection and prevent systemic infection. Extracellular vesicles (Evs) act as messengers between macrophages infected with *C. albicans* and uninfected macrophages. When macrophages are infected with *C. albicans*, they secrete more Evs, migrate extracellularly, and activate peripheral or circulating monocyte ERK 2 and β 38 enzymes, which are inflammatory factors that enhance the ability to kill *C. albicans* (Liu et al., 2019).

##### Effect of the M1 and M2-type macrophages on *Candida albicans*

Macrophages can be divided into classically activated inflammatory macrophages (M1) and alternatively activated anti-inflammatory macrophages (M2). M1 is polarized in an inflammatory environment to produce proinflammatory cytokines, whereas M2 is anti-inflammatory and contributes to tissue repair during wound healing. Bacterial endotoxin (lipopolysaccharide; LPS) is an effective factor in infection that induces M1 to produce higher levels of iNOS, TNF α, and IL -12p70, thereby determining the inflammatory T cell response. M2 can be converted into M1 macrophages after LPS stimulation to promote inflammation. It has been shown that heat-killing *C. albicans* (HKC) strongly suppresses LPS-induced IL -12p70 production in M2 macrophages (Zheng et al., 2013). *C. albicans* induces the production of the anti-inflammatory cytokine IL -35 in M2 and blocks the LPS-induced conversion of M2 to the M1 phenotype. During the resolution phase of infection and wound healing, M1 can be polarized to M2 in tissues. M2 produces lower levels of inflammatory cytokines but higher levels of anti-inflammatory cytokines and growth factors after HKC stimulation. However, sustained production of inflammatory cytokines induced by M1 may lead to persistent inflammation caused by excessive Th1 and Th17 responses. Although the immigration of numerous Th1 and Th17 cells into the inflamed tissue contributes to increased killing by macrophages, they may also cause tissue damage. Combating pathogen invasion and initiating tissue repair by increasing concentrations of growth factors and releasing anti-inflammatory cytokines into tissues is associated with M2. M2 also exhibits phenotypic and functional plasticity, such as LPS, a potent bioactive factor that can cause macrophage phenotype transition from M2 to M1.

### *Candida albicans* mediates macrophages immune tolerance

*C. albicans* is present on the moist mucosal surfaces of most healthy individuals and does not cause disease, and this symbiotic presence is associated with host immune tolerance. There are two basic morphological growth forms of *C. albicans*, hyphae and yeast. Mycelia on the mucosal surface elicit an immune response, whereas the primary presence of yeast is associated with symbiotic presence on the mucosal surface and may be more effective in inducing immune tolerance. IL-34 is able to promote the conversion of M1 to M2, which may benefit the skin in establishing immune tolerance and wound healing (Diez-Orejas et al., 2018b). *C. albicans* can inhibit host inflammatory responses in the skin mucosa by inhibiting LPS-induced IL-12p70 production, while lower IL-12p70 production can avoid unnecessary Th1 responses to maintain immune tolerance, which may be one of the mechanisms by which *C. albicans* achieves a successful symbiotic lifestyle without compromising host health (Shao et al., 2022). IL-12p70 is an important proinflammatory cytokine that determines Th1 polarization, inhibits LPS-induced IL-12p70 production, and may be a key mechanism of *C. albicans*-induced immune tolerance. During the clearance of infection that promotes wound healing, proper conversion of macrophages from the M1 to the M2 phenotype is critical to limit tissue inflammation and promote tissue healing. Maintaining the M2 phenotype is key to maintaining immune tolerance. *C. albicans* induces the expression of EBI3 in M 2 and blocks the conversion of M2 to M1 phenotype induced by LPS, which may also be one of the mechanisms by which *C. albicans* induces immune tolerance (Patel, 2022).

### *Candida albicans* mediates macrophages immune escape

Traditionally, masking of fungal antigenic ligands has been viewed as a strategy of fungal immune evasion in invasive infections (Pellon et al., 2022). However, In the process of interaction between *C. albicans* and macrophages, it avoids the killing effect of macrophages by blocking the recognition by macrophages, inhibiting the maturation of phagosome or neutralizing the pH of phagosome, changing the properties of macrophages and inhibiting the sterilization, lytic output or non-lytic export pathway to change its morphology or metabolic reprogramming.

#### Blocking of macrophages recognition

*C. albicans* is ability to form biofilms and hyphae, produce hydrolytic enzymes and candidiasis. Although mucosal immunity is activated, the combination of increased abundance and virulence of this pathogenic organism leads to infection, first through the formation of mycelial associated toxins by colonizing *Candida albicans* cells (Deng et al., 2019). Clinically refractory *C. albicans* infections suggest that the physical structure of the biofilm impedes macrophage migration, limiting macrophage antimicrobial activity and making the biofilm a host for persistent infection. The *C. albicans* surface amyloid and human binding serum amyloid β-component (SAP) impair recognition by macrophages and inhibit the macrophage immune response. Svoboda et al. (2015) found that *C. albicans* can produce secreted aspartyl proteinase 2 (Sap2)-cleaving complement inhibitor (FH), reduce the amount of FH recognized by CR3 and CR4, and impair recognition and killing by macrophages. In inducing oxidative stress, *C. albicans* promotes β-mannosylation of cell wall components, reduces hydrophobicity on the cell surface thereby decreasing ERK1/2P levels in macrophages, promotes oxidation and TNF-α production, and increases resistance of the fungus to macrophages (Ibata-Ombetta et al., 2003).

#### Inhibition of phagosomal maturation or neutralizing phagosomal pH

*C. albicans* is recognized, endocytosed by macrophages, and then trapped in phagosomes. These phagosomes are remodeled to obtain antimicrobial substances and lysis enzymes, e.g., by membrane fusion to acidify the phagosomal lumen. For *C. albicans*, which is engulfed by macrophages, to survive, it must destroy the powerful bactericidal machinery in the phagolysosomes.

(1) Inhibition of phagosome maturation.

Type O-mannose masks the p-glucan in the inner layer of the cell wall and suppresses the recognition of dectin-1, which is involved in promoting phagosome maturation. Strains lacking O-mannose in the cell wall increase the ability of mature phagosomes to bind RabGTPase and inhibit the growth of phagocyte filaments. *C. albicans* slows phagosome maturation by hyphal elongation.

(2) *C. albicans* neutralizes the pH of phagosomes by physical disruption or metabolic reprogramming.

The importance of metabolism and nutrient availability in fungus-host interactions has been highlighted in recent years. Upon activation, immune cells and other host cells reshape their metabolism to meet the energy-demanding processes that generate an immune response. These include up-regulation of glucose uptake by macrophages and treatment by aerobic glycolysis. Candida, on the other hand, ADAPTS its metabolic pathways to normally hostile environments in the host, such as the lumen of the phagosome. Further understanding of metabolic interactions between host and fungal cells may lead to new/enhanced antifungal therapies to combat these infections (Pellon et al., 2022). *C. albicans* disrupts the integrity of phagosomal membranes through hyphal growth, communicates phagosomes with the cytoplasm, neutralizes phagosome pH, causes morphological changes in hyphelias, and promotes survival within hyphal cells. After phagocytosis of *C. albicans* by macrophages, genes involved in arginine biosynthesis are upregulated, and arginine is converted to urea and degraded to produce CO2 and NH3, neutralizing acidic conditions and promoting hyphal growth. At the same time, *C. albicans* can utilize pyruvate, α-ketone, glutaric acid and lactic acid as the main carbon sources to neutralize the acidic environment rapidly.

#### Change the macrophages properties

Normally, *C. albicans* is a commensal bacterium that is harmless to the skin and mucous membranes. *C. albicans* modulates the antimicrobial activity of macrophages by altering their properties. During *C. albicans* infection, macrophages synthesize nitric oxide via nitric oxide synthase (iNOS) and kill invading pathogens. *C. albicans* can produce extracellular DNA enzymes that are thought to degrade DNA, a structural component of METs, to prevent death of Macrophages and cause M1 to M2 type conversion, thereby increasing *C. albicans* survival.

#### Dissolution or non-dissolved output pathway

The dissolution-release pathway is the process by which *C. albicans* causes death and rupture of macrophages through morphological and metabolic changes, releasing phagocytosed *C. albicans* and causing disseminated infection. The febrile pathways caused by hyphal growth, nutrient starvation, and cell death are responsible for the lysis and release of macrophages (Deng et al., 2019). *C. albicans* can utilize multiple carbon sources in the phagocytosis of macrophages to increase its resistance in the presence of glucose. Carbon sources can induce drug resistance to fluconazole, and *C. albicans* undergoes a continuous transcriptional reprogramming process after phagocytosis by phagocytes. At an early stage, activation of the gluconeogenesis pathway and fatty acid depletion result in starvation of cells, and the glycolysis pathway is restored when *C. albicans* is engulfed. During the interaction between *C. albicans* and macrophages, metabolic changes are triggered between them that can enhance the glycolytic pathway and lead to glucose competition. *C. albicans* relies on various carbon sources for its intracellular growth, but infected macrophages can survive only with the help of glucose in glycolysis. *C. albicans* rapidly consumes glucose, and macrophages are killed due to lack of energy supply.

## Effect of *Candida albicans* virulence on macrophages

The morphological transition between yeast and hyphal forms of *C. albicans*, expression of cell surface adhesins and invasins, tropism, biofilm formation, phenotypic turnover, and secretion of hydrotolytic enzymes are considered virulence factors. The pathogenic yeast *C. albicans* was found to be coated with phospholimannose (PLM) consisting of a β-1,2-oligomannose site and phytoceramide. PLM-induced externalization of membrane phosphatidylserine, loss of mitochondrial integrity, and DNA fragmentation suggest that PLM promotes yeast survival by inducing macrophage death (Ibata-Ombetta et al., 2003).

## Summary and outlook

Interaction of *C. albicans* with macrophages is an important immune system defense response to Candida disease associated with deep dissemination of Candida cells in humans. Despite the obvious efficiency of killing pathogens, macrophages fail to effectively control the disease process of candidiasis in severely damaged patients. The patients with low immunity, prolonged use of antibiotics and immunosuppressants may develop severe *C. albicans* infection. The development of drugs against the transformation of the *C. albicans* form and virulence-related target drugs has become an effective means for the accurate diagnosis and treatment of *C. albicans*. On the other hand, macrophage remodeling promotes macrophage redifferentiation from M2 to M1 phenotype, which promotes macrophage phagocytosis and lysosomal digestion of *C. albicans*. In conclusion, our study investigated the mechanism behind the escape of *C. albicans* hyphae from macrophages. Inhibition of hyphal escape can reduce the inflammatory response of macrophages to *C. albicans* infection and provides an accurate prevention, diagnosis, and treatment basis for *C. albicans* infection.

## Data availability statement

The original contributions presented in this study are included in the article/supplementary material, further inquiries can be directed to the corresponding author/s.

## Author contributions

SZ, AS, and XH designed the research study. MG, LS, and YH carried out the data analysis and processing. SZ prepared the original manuscript. AS and XH revised the manuscript and edited the final version. All authors read and approved the final manuscript.
